# Multiple teeth replacement with endosseous one-piece yttrium-stabilized zirconia dental implants

**DOI:** 10.4317/medoral.18194

**Published:** 2012-08-28

**Authors:** Andrea E. Borgonovo, Alberto Fabbri, Virna Vavassori, Rachele Censi, Carlo Maiorana

**Affiliations:** 1 DMD, MD. Clinical Assistant Professor, School of Oral Surgery , University of Milan, Italy; 2 DDS. DDS Department of Implantology, Dental Clinic, Fondazione IRCCS Ca’ Granda Ospedale Maggiore Policlinico, Milan, Italy; 3 DDS. Associate Professor, School of Oral Surgery , University of Milan, Head Department of Implantology, Dental Clinic, Fondazione IRCCS Ca’ Granda Ospedale Maggiore Policlinico, Milan, Italy

## Abstract

Objectives: The purpose of this study is to clinically and radiographically evaluate survival and success rate of multiple zirconia dental implants positioned in each patient during a follow-up period of at least 12 months up to 48 months. Study Design: Eight patients were treated for multiple edentulism with 29 zirconia dental implants. All implants received immediate temporary restorations and 6 months after surgery were definitively restored. 6 months to 4 years after implant insertion, a clinical-radiographic evaluation was performed in order to estimate peri-implant tissues health and peri-implant marginal bone loss. Results: Survival rate within follow-up period was therefore 100%. The average marginal bone loss (MBL) from baseline to 6 months was +1.375±0.388 mm; from 6 months to 1 year was +0.22±0.598 mm; from 1 year to 2 years was -0.368±0.387 mm; from 2 years to 3 years was -0.0669±0.425 mm; from 3 years to 4 years +0.048±0.262 mm. The mean marginal bone loss at 4 years from the implants insertion was +1.208 mm. Conclusions: According to several studies, when using a radiographic criterion for implant success, marginal bone loss below 0.9-1.6 mm during the first year in function can be considered acceptable. In our work, radiographic measurements of MBL showed values not exceeding 1.6 mm during the first year of loading and also 1 year up to 4 years after surgery further marginal bone loss was minimal and not significant. This peri-implant bone preservation may be associated to the absence of micro-gap between fixture and abutment since zirconia dental implants are one-piece implant. Moreover, zirconia is characterized by high biocompatibility and it accumulates significantly fewer bacteria than titanium.

** Key words:**Zirconia dental implants, multiple implants, radiographic evaluation, marginal bone loss (MBL).

## Introduction

Pure titanium is the best material for endosseous dental implant since Branemark et al. (1) reported high success rate with the direct bone-implant interface termed osseointegration. This biocompatible material has been used for about 30 years as implant substrate and it has shown high success rates. However, the gray colour of the titanium may be disadvantageous and give rise to esthetic problems, especially if the soft tissue situation is not optimal and the dark colour shines through the thin peri-implant mucosa (2). A possible alternative to titanium might be tooth-coloured materials such as ceramics (3). One ceramic material that was used in the past for dental implants was aluminium oxide (4). This material showed good osseointegration, but it did not have sufficient mechanical properties for long-term loading and it was withdrawn from the market. Partially stabilized zirconia (PSZ), which is comparable to the highest values of bending strength for oxide ceramics (5), has been introduced as a new bioinert ceramic implant material. The PSZ possesses more favorable mechanical properties than the fully stabilized zirconia, and has high fracture toughness because of its energy-absorption property and it behaves like steel (6). Zirconia in fact presents high flexural strength (900-1200 MPa), hardness (1200 Vickers) and Weibull modulus (7,8). Furthermore, its biocompatibility, as a dental implant material, has been demonstrated in several investigations (8-10). In vitro simulations showed that the material appears to be capable of withstanding loads over the long term (11).

The aim of our work is to clinically and radiographically evaluate survival and success rate (12) of multiple zirconi dental implants positioned in each patient during a follow-up period of at least 12 months up to 48 months.

## Material and Methods

-The aim of this study was to:

- Evaluate survival rate of multiple endosseous one-piece yttrium-stabilized zirconia dental implants, positioned in each patient (White-SKY® Bredent,Senden Germany) in a follow-up period of 12-48 months after insertion.

- Evaluate peri-implant soft tissues health and radiographic bone remodeling after at least 6 months from definitive restoration positioning.

-Implant System

White-SKY® (Bredent,Senden Germany) implants are fabricated in Brezircon®, a zirconium dioxide tetragonal polycrystal ce-ramic, hot isostatically pressed, anallergic, biocompatible, with high flexural strength (1250 MPa).White Sky® is a one-piece implant, with a design characterized by a conical body with double threads and rounded apex. The endosseal portion has a sandblasted surface, whereas in the gingival region the implant features a machined neck with a height of 2 mm. The abutment surface is also machined, has a length of 6.8 mm and can be customized by grinding after the insertion. The implant surface is treated with a sanding process. The microscopical surface characteristics of medium rugosity (Ra 0.9-1 m) are similar with the surface of last-generation machined-finished titanium implants.

-Study design and inclusion criteria

From September 2007 to January 2011, 8 patients in need of multiple teeth replacement both in maxillary and mandibular arch were selected for this study. Patients presented each multiple edentulism involving different areas of both jaws. In case of multiple edentulism every missing tooth was substituted by an implant except long edentulous span where pontic was programmed. All implant sites should present adequate bone volume (height >8mm, thickness > 5.5 mm). Patients with total edentulism and implants positioned in regenerated bone were excluded from this protocol. Patients aged < 18 years, with previous or concomitant systemic diseases such as immunodeficiency, head and neck radiotherapy, metabolic disorders, hematological diseases, bisphosphonates treatment, smoking > 10 cigarettes a day, with poor oral hygiene and low compliance were not included in this study. To be included in this protocol moreover, all patients should not present oral problems such as active periodontal disease and parafunctions.

Patients were previously informed about zirconia implants and possible alternatives, and gave their written consent. Prior to surgery, an orthopantomography or standardized periapicall radiography was obtained. According to this protocol, standardized periapical radiography using the Rinn alignment system with silicone bite, should be taken 1 week, 6 months, 12 months, 24 months, 36 months and 48 months after implants insertion.

Mesial and distal marginal bone levels of all implants were determined at baseline and recall evaluations. Measurements were obtained from images of successive radiographs, which were scanned and digitized (Epson 1680 Pro, Seiko Epson Cooperation, Nagano, Japan) before, and analyzed at x20 magnification using a software program (CorelDraw 10; Corel Corp and Coral Ltd, Ottawa, Canada). The known length of the implant (measured from the implant shoulder to the implant apex) according to the manufacturer’s dimensions of the respective implants was used as reference point. The distance from the implant shoulder to crestal bone level was measured on the magnified images. To account for variability, the implant dimension (length) was measured and compared to the documentation dimensions; and ratios were calculated to adjust for distortion. Bone levels were determined by applying a distortion coefficient. The actual bone level measurement was performed independently by 2 examiners. The average from both examiner calculations was used as marginal bone level value. The level at which the marginal bone seemed to be attached was assessed by visual evaluation at the distal and mesial surfaces of all implants. Data analysis was performed with descriptive statistics and the mean and standard deviation were calculated.

**Figure 1:**
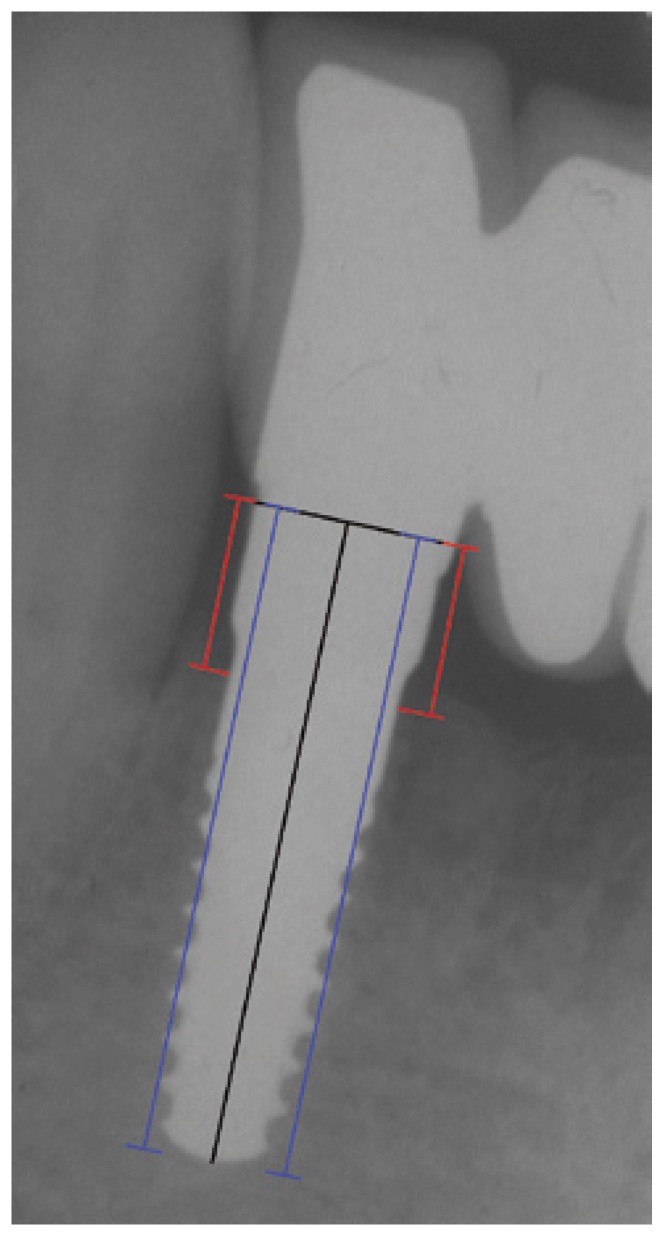
Twenty-four months after surgery: radiographic control of a zirconia dental implant positioned in 4.2. Assessment of the distance from the shoulder to the first bone-to-implant contact on digitalized radiographs (red lines). The blue lines refer to implant length.

For all patients impressions were taken to obtain casts for wax-up in order to fabricate surgical splint and provisional restorations. (Fig. 1)

-Surgical protocol

All patients underwent professional oral hygiene 7 days before surgery and were instructed to start rinsing mouth twice a day with chlorhexidine 0.2% (Corsodyl®, Glaxo UK) until 2 weeks after surgery. Antibiotic prophylaxis with amoxicillin and clavulanic acid (Laboratori Eurogenerici, Milano Italy) was prescribed 1 hour prior to surgery and therapy continued with 1g every 8 hours for 7 days. The surgical protocol was followed as suggested by Bredent Medical® and similar to standard surgical protocol for titanium implants. A mucoperiosteal flap was elevated before implant site drilling, and when implant site presented fenestrations or dehiscences, regenerative procedures by resorbable membranes (Biogide® Geistlich Pharma AG Wolhusen, Switzerland) and bone substitutes (Bio-oss® Geistlich Pharma AG Wolhusen, Switzerland) were performed. A surgical guide obtained by wax up on pre-operatory casts was used in all cases to achieve implants’ optimal position and inclination. Implant site preparation was performed in order to leave implant abutment with smooth neck heal transmucosally, whereas implant body rough surface was left completely inside the bone. Flaps were sutured with 4/0 monofilament suture (Premilene® ,Braun Melsungen Germany). When necessary before suturing flaps, periosteal releasing incisions were performed to attain primary wound closure. Patients were given oral hygiene suggestions and were instructed not to chew or eat on implant site until healing was completed and to continue with antibiotic therapy and chlorhexidine mouth-rinses and use of analgesic, Paracetamol 500mg, (Tachipirina®, Angelini ,Roma Italy ) if necessary.

**Figure 2:**
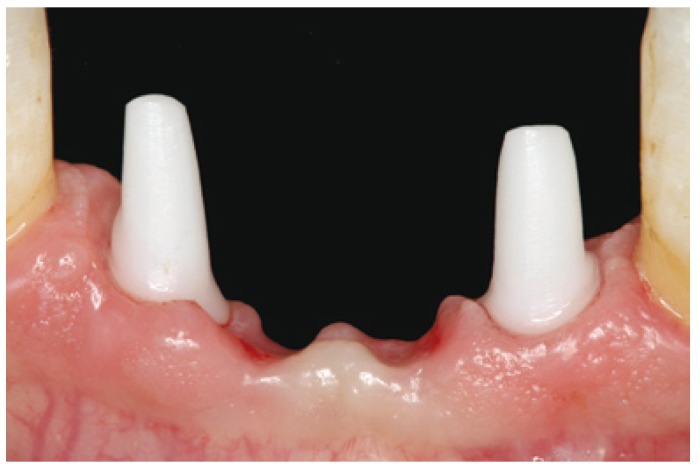
Soft tissue healing 6 months after surgery.

Sutures were removed 7 days after surgery. Follow-up controls were programmed after 1 week, 2 weeks and subsequently once a month for the following 6 months. (Fig. 2)

-Immediate Temporary restoration protocol

Immediately after implant insertion, implant abutment is prepared in order to correct its axis, length or undercuts if present, using double diamond burs suited for zirconia (ETERNA®, BREDENT, Senden, Germany) and water cooling. Since zirconia ceramics are not termic conductors this procedure does not allow for the risk of bone necrosis for overheating. After that, temporary restoration obtained from diagnostic wax up is relined with acrylic resin on temporary prosthetic caps positioned on implant abutments, thus preventing gingival tissue from growing over implant abutment and allowing a greater precision of temporary restoration margins on abutment finishing-line shoulder. Following occlusal controls in order to leave restorations not subjected to functional load and to avoid lateral contacts, the provisional restorations are luted with temporary cement (TEMP BOND®, Kerr West Collins Orange CA). Multiple implants are connected together by provisional restoration, in order to reduce the risk of implant mobility or extra-occlusal load (tongue and lips movements).

All patients were visited 1 week, 2 weeks and once a month after surgery, in order to control implant stability, peri-implant soft tissues health and restorations integrity. After 6 months from implant insertion implant abutment length or axis were furtherly corrected when necessary and definitive impressions (IMPREGUM, 3M, ESPE, St Paul, MN) were taken after using a retraction cord (Ultrapak® Cord, ULTRADENT, South Jordan, UT) or impression caps to register implant abutment shoulder margins. Definitive all-ceramic zirconia crowns or bridges were made with CAD-CAM system (LAVA; 3M ESPE, St Paul, MN) and cemented with a glassionomer cement (GC Fuji CEM, GC America, Alsip IL).

**Figure 3:**
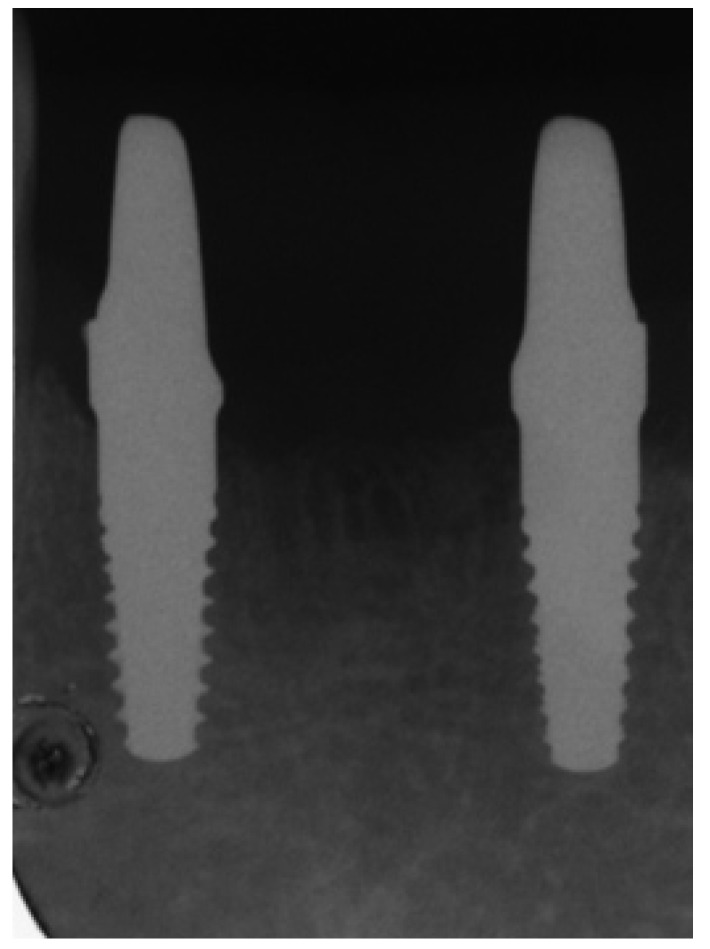
Radiographic control 6 months after implant insertion.

6 months after definitive restorations were cemented (at least 12 months after implant insertion) clinical-radiographic evaluation (Fig. 3) was performed as follows:

- Periodontal evaluation with calibrated probe (Hu-Friedy®, N. Rockwell Chicago IL,)

- Bleeding on probing (BOP): 0 = negative, 1 = positive after superficial probing, 2 = spontaneous

- Pocket depth

- Plaque index: 0 = plaque not present, 1 = plaque present

- Mobility: 0 = implant stability, 1 = implant mobility 

- Peri-implant marginal bone loss resorption radiographically evident

Success criteria were formulated according to the following parameters:

- Lack of mobility

- Absence of self-reported pain or paresthesia 

- Lack of peri-implant radiolucency 

- Peri-implant marginal bone loss inferior to 1.5 mm in the first year and 0.2 mm in the following years.

Survival criteria (12) were identified as the survival of loaded functionalized asynthomatic implants. These recordings were repeated 1 year after delivery of definitive restorations.

## Results

In the department of Implantology , Dental Clinic IRCCS Foundation Hospital, University of Milan, 8 patients were treated for multiple edentulism with 29 one-piece Yttrium partially stabilized zirconia dental implants. Two of these patients, with 9 implants placed, were excluded from the study because the implants were inserted in regenerated bone. It has been shown that the marginal bone loss is greater in regenerated bone than the native bone (13). One of these patients, with 2 implants placed, was excluded from the study because he did not attend to follow-up. Mean age of the 5 patients, with 18 implants placed, was 56.25 years (range 50 to 65 years). 20 implants were positioned for the treatment of multiple edentulism, the follow up period ranged from 12 to 48 months from implant insertion. All implants were positioned in native bone. Six implants were placed in mandible and 14 implants in maxilla.

Mean implant diameter was 4 mm and mean implant length was 12 mm. All implants were immediately restored with a provisional prosthesis. All multiple implants were splinted together by provisional restoration. No implant failure was reported during the 48 months follow-up. All implants received definitive restoration 6 months (8 months in 5 implants where regenerative procedures had been performed in the same time of the implant insertion).

In accordance to Albrektsson criteria (12), survival rate within follow-up period is therefore 100%. Six months after definitive restorations, periodontal indexes were registered ( Table 1) for each patient at each implant site (plaque index, bleeding on probing, probing pocket depth in 6 different points for each implant, mobility) and standardized radiographs were taken and compared to radiographs taken immediately and 6 month post-surgery. Plaque index resulted 1 for 10 implants (50%) and 0 for 10 implants (50%). Bleeding on probing index resulted 1 for 10 implants (50%) and 0 for 10 implants (50%). Five implants presented at least 1 point with PD ranging from 3 to 5 mm (25%). No implants presented PD values > 5 mm. Mobility was not present at any site. No pain (spontaneous or on percussion) and paresthesia were reported. Radiographic evaluation reported 100% absence of peri-implant radiolucency. Marginal bone loss radiographically evident resulted >1.5 mm only in 2 sites 6 months after definitive prosthetic restoration. The average margi-nal bone loss from baseline to 6 months was +1.375±0.388 mm; from 6 months to 1 year was +0.22±0.598 mm; from 1 year to 2 years was -0.368±0.387 mm; from 2 years to 3 years was -0.0669±0.425 mm; from 3 years to 4 years +0.048±0.262 mm. The mean marginal bone loss at 4 years from the implants insertion was 1.208 mm.

**Table 1:**
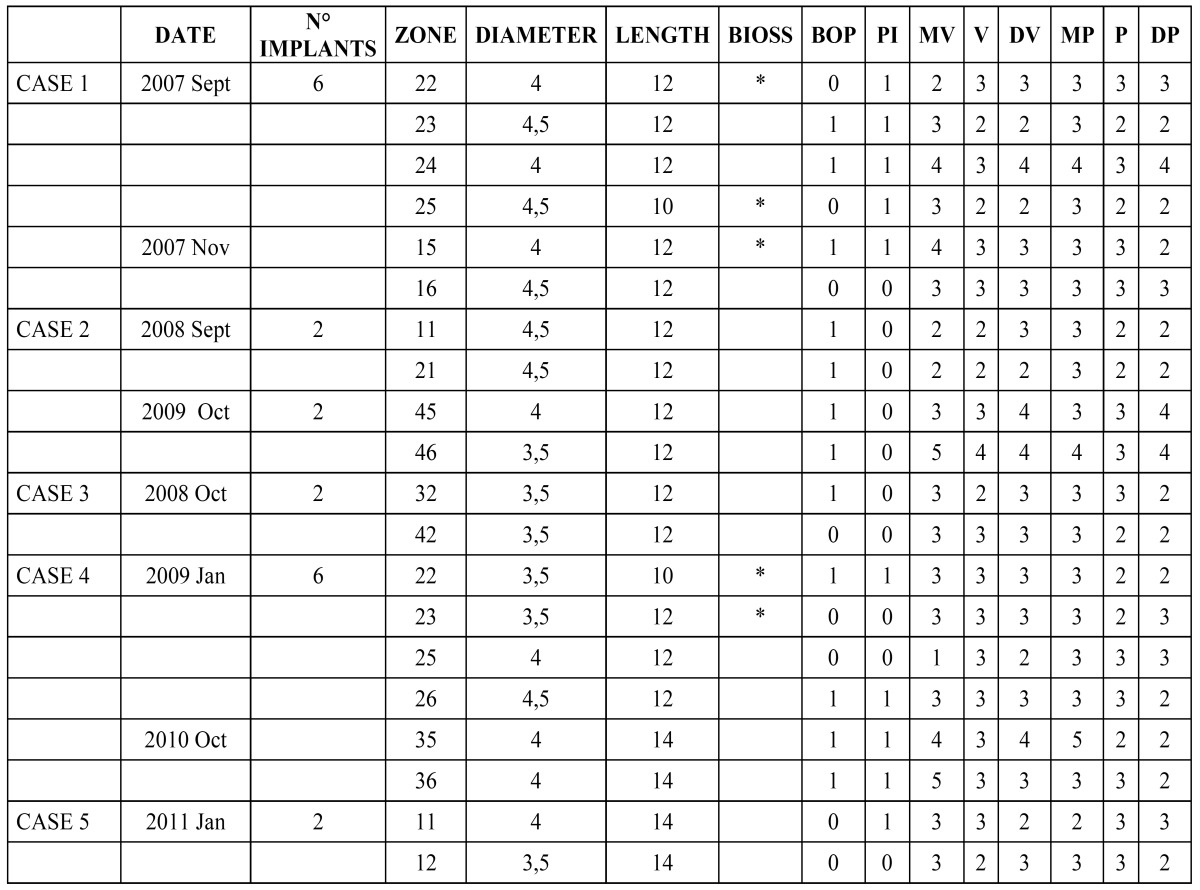
Results of the clinical measurements, 6 months after final prosthesis delivery.

For all implants, periodontal and radiographic recordings were repeated 18 months after implant positioning, and the results were substantially stable even if these data have not completely been elaborated yet.

## Discussion

Osseointegration of threaded zirconia implants has been demonstrated in various animal models. Akagawa et al. (14) compared the bone tissue response to loaded and unloaded zirconia implants in the dog mandible. The authors reported high degrees of bone-implant contact 3 months after implantation, with no significant difference between the groups. Scarano et al. (15) investigated the bone response to 20 YTZP implants, which were inserted in the tibiae of five rabbits. According to the authors, all implants were osseointegrated without signs of inflammation or mobility. The mean bone implant contact (BIC) was calculated to be 68%. In a monkey model, Akagawa et al. (9) examined the possibility of long-term stability of osseointegration around partially stabilized zirconia implants placed in a 1-stage procedure with single freestanding implant support, connected freestanding implant support, or a combination of implant and tooth support. The animals were sacrificed after 12 and 24 months. The authors’ hypothesis was that the continuous loading and the type of loading might influence bone-implant interface. The results obtained failed to show statistically significant differen-ces, but it was observed that after 12 months single freestanding implants showed bone-implant contact inferior to the other two groups. The authors hypothesized that during early osseointegration phases lateral forces from uncontrolled parafunctions such as tongue movements may influence healing and thus concluded that implant connection is more favorable to bone-implant interface stability.

The results of the present study have shown a reduction of marginal bone loss around zirconia implants because it seems that splinted prostheses generated more uniform strain distributions respect of non-splinted implant prostheses (16). The remodeling process involves marginal bone resorption that is affected by one or more of the following factors (17-21): 1) a traumatic surgical technique; 2) excessive loading conditions; 3) the location, shape, and size of the implant abutment micro-gap and its microbial contamination; 4) the biologic width and soft tissue considerations; 5) a peri-implant inflammatory infiltrate; 6) micromovements of the implant and prosthetic components; 7) repeated screwing and unscrewing; 8) the implant-neck geometry; and 9) the infectious process. The values generally accepted as a reasonable guideline for bone loss since the late 80’s is -1.5 mm for the first year post-loading of the implants and -0.2 mm of additional loss for each following year (12,22). On the basis of clinical observations, bone loss ranging between 1 and 2.6 mm has been reported to occur around the margin of successfully osseointegrated dental implants (23,24). There are studies reporting bone gain or no bone changes for individual implants, aside from bone resorption (25,26). Mean annual losses of 0.03 to 0.05 mm were reported for ITI implants (24,26). The results of this study showed around zirconia implants, between baseline and 6 month, a mean marginal bone loss of -1.375 mm, between 6 months and 1 year -0,4275 mm, between 1 year and 2 years 0,239 mm and between 2 years and 3 years –0.04625 mm. It is interesting to observe a reduction of marginal bone level during the first 6 months and then a stability of this level over time, up to 4 years.

According to the authors, the reduction of marginal bone is mainly due to the one-piece morphology of zirconia dental implants through which there is no implant-abutment micro-gap and its microbial contamination, there are not micro-movements of the prosthetic component and repeated screwing and unscrewing. Numerous studies have shown that bone resorption around the implant neck does not start until the implant is uncovered and exposed to the oral cavity. This invariably leads to bacterial contamination of the gap between the implant and the superstructure (27). Bone remodeling will progress until the biologic width has been created and stabilized. Not only does this width progress apically, along the vertical axis, but according to studies conducted by Tarnow et al (28), there is also a horizontal component amounting to 1-1.5 mm. Another factor, according to the authors, led to a reduction of marginal bone loss is the reduction of bacteria on the surface of zirconia. Scarano et al. (29) compared bacterial adhesion between zirconium oxide and titanium surfaces. Zirconium oxide surfaces showed a significant reduction of the presence of bacteria, and this fact is probably important for the health of the peri-implat soft tissues. A bacterial adhesion to implant surfaces is a first stage of peri-implant mucositis and peri-implantitis; in fact a positive correlation has been found between oral hygiene and marginal bone loss around implants (30). The reduction of bacterial adhesion on the surface of zirconia implants promotes early formation of the biologic width and therefore the formation of a mucosal seal that stops early marginal bone resorption.
